# The Climate-Driven Genetic Diversity Has a Higher Impact on the Population Structure of *Plasmopara viticola* Than the Production System or QoI Fungicide Sensitivity in Subtropical Brazil

**DOI:** 10.3389/fmicb.2020.575045

**Published:** 2020-09-17

**Authors:** Ricardo F. Santos, Maisa Ciampi-Guillardi, Bart A. Fraaije, Amanda A. de Oliveira, Lilian Amorim

**Affiliations:** ^1^Department of Plant Pathology and Nematology, Luiz de Queiroz College of Agriculture, University of São Paulo, Piracicaba, Brazil; ^2^National Institute of Agricultural Botany, Cambridge, United Kingdom; ^3^Department of Genetics, Luiz de Queiroz College of Agriculture, University of São Paulo, Piracicaba, Brazil

**Keywords:** grapevine, downy mildew, quinone outside inhibitor, population genetics, microsatellite markers, oomycete reproductive strategy

## Abstract

Downy mildew, caused by *Plasmopara viticola*, is the main disease affecting vineyards in subtropical Brazil. Here, we collected 94 *P. viticola* isolates from four organic and conventional vineyards in the two main grape-growing states of Brazil to evaluate the sensitivity to the quinone outside inhibitor (QoI) azoxystrobin by pheno- and genotyping assays. The impact of location, production system and sensitivity to QoI fungicides on the population genetics and structure of *P. viticola* was determined using 10 microsatellite markers. Cytochrome *b* sequencing revealed that 28 and 100% of the isolates from vineyards under organic and conventional management carried the G143A mutation, respectively. The G143A mutation was associated with high levels of azoxystrobin resistance. Three out of the 94 isolates analyzed carried the M125I alteration, not previously described in *P. viticola*, which was associated with a five-fold reduction in azoxystrobin sensitivity compared to wild-type isolates. Haplotype network analysis based on cytochrome *b* gene sequences suggested that the Brazilian populations are more closely related to the European than the North American population. A total of six haplotypes were identified, with two of them carrying the G143A mutation. Microsatellite analysis revealed high allelic and genotypic variation among the four populations. Population differentiation analyses indicated that state of origin directly influences the population biology of *P. viticola*, while production system and QoI sensitivity have little effect. Great genetic diversity, sexual reproduction and high levels of admixture were observed in Rio Grande do Sul State. In contrast, populations in São Paulo State were dominated by a few clonal genotypes, and no admixed genotype was detected between the two genetic pools identified in the state. This study raises the hypothesis that winter weather conditions influence the overwinter survival strategy with profound effects in the population biology of *P. viticola*.

## Introduction

Downy mildew, caused by *Plasmopara viticola*, is considered one of the most destructive diseases of grapevine worldwide. Under favorable weather conditions and absence of control measures, the pathogen can infect all green parts of the grapevine causing losses up to 100% (Caffi et al., 2011). The pathogen is native to North America and was accidentally introduced into the main grapevine-growing regions of Australia, Brazil, South Africa and Europe by infected propagation material at the end of the 19th and the beginning of the 20th centuries (Dewar, 1907; de Castella and Brittlebank, 1917; Sousa, 1996; Agrios, 2005). After the introduction in Brazil, *P. viticola* successfully spread and sustained itself across Southern and South-eastern regions, where the subtropical climate is characterized by frequent rain and average daily temperatures between 20 and 27°C during the grape-growing season (Amorim et al., 2016).

*Plasmopara viticola* is a biotrophic, diploid and heterothallic oomycete with two mating types, P1 and P2 (Wong et al., 2001; Gessler et al., 2011). In temperate regions, the pathogen overwinters as oospores in leaf debris on the vineyard floor. During the spring, oospores germinate and produce sporangia that release zoospores, which are responsible for primary infections. After 5 to 6 days at 18 to 26°C, the pathogen produces new sporangia on the lesions that cause secondary infections (Gobbin et al., 2006; Kassemeyer et al., 2015). Nevertheless, an overlap in the availability of primary and secondary inoculum can occur during part (Rossi et al., 2008) or even throughout the season (Rumbou and Gessler, 2004; Kennelly et al., 2007). In contrast to temperate regions where the *P. viticola* biology is well-documented, little is known in subtropical regions. In Southern Brazil, the temperatures during the winter are low enough to induce grapevine bud dormancy and complete leaf fall that may promote mating and oospore formation, serving as a survival strategy. Meanwhile, the climatic conditions in South-eastern Brazil are favorable for grapevine growth all-year-round, allowing two harvests a year through double pruning management (Pommer, 2006). The double cropping system increases yield significantly, but may serve as a green bridge for pathogens, where *P. viticola* appears to survive predominantly asexually between seasons (Camargo et al., 2019).

Control of grapevine downy mildew is mainly based on multiple fungicide applications throughout the growing season since most of the commercial cultivars are moderately to highly susceptible (Kassemeyer et al., 2015). In organic vineyards, downy mildew is controlled by regular sprays with copper compounds (Dagostin et al., 2011), while phenylamide, quinone outside inhibitor (QoI) and carboxylic acid amide (CAA) among other fungicide groups are extensively used in conventional viticulture (Gisi and Sierotzki, 2015; Delmas et al., 2017; Toffolatti et al., 2018). However, repeated applications of single-site fungicides can select and increase the frequency of pre-existing or evolving genotypes carrying alleles encoding resistance to fungicides in the population (Milgroom, 2015). For instance, applications of QoIs throughout one season reduced the genetic diversity of a Swiss *P. viticola* population, as a consequence of strong directional selection toward QoI resistance (Matasci et al., 2008).

The QoIs inhibit mitochondrial respiration by blocking electron transfer at the cytochrome-*bc*1 enzyme complex disrupting energy production, with a strong inhibitory activity against oomycetes and fungi as result (Bartlett et al., 2002). In *P. viticola*, QoI resistance is conferred by the replacement of glycine by alanine at codon 143 (G143A) in cytochrome *b*, which is associated with high resistance levels (Chen et al., 2007). A second amino acid substitution at codon 129 (phenylalanine to leucine) has rarely been observed (Sierotzki et al., 2005). Phylogenetic analysis indicated that the A143 resistant allele appeared independently twice in Europe (Chen et al., 2007; Delmas et al., 2017). Although QoIs have been used in viticulture around the world for more than two decades (Furuya et al., 2010), there is limited knowledge on QoI resistance in *P. viticola* in Brazil.

Population genetic analyses are useful tools to understand how pathogens emerge and adapt to different geographical areas as well as crop management practices (McDonald and Linde, 2002; Milgroom, 2015; Grünwald et al., 2017). Previous studies, mainly conducted in Europe using microsatellite markers, revealed random mating and high levels of allelic and genotypic diversity in *P. viticola* (Gobbin et al., 2006; Delmas et al., 2017). Although downy mildew has been causing losses in Brazilian vineyards for over a century (Sousa, 1996), the genetic structure of *P. viticola* populations has been only recently investigated in fungicide-treated vineyards from South-eastern region (Camargo et al., 2019). In this study, we examined whether production system, QoI sensitivity and geographical origin have an influence on *P*. *viticola* population diversity, genetic structure and reproduction. We also investigated the sensitivity of *P. viticola* to the QoI azoxystrobin, using pheno- and genotyping assays, and the relationship with different cytochrome *b* haplotypes.

## Materials and Methods

### *Plasmopara viticola* Sampling and Isolation

Leaves showing typical symptoms of downy mildew were collected from four commercial vineyards, two located in São Paulo State (PPv1 and PPv2), South-eastern Brazil, and two in Rio Grande do Sul State (PPv3 and PPv4), Southern Brazil, in the 2018/19 growing season. Two locations consisted of vineyards under organic production system for at least six years (PPv1 and PPv4), where only copper and sulfur based products were used to control foliar diseases. The other two vineyards (PPv2 and PPv3) were cultivated under conventional production system, where disease control was based on application of multi- and single-site fungicides throughout the growing season. Distances between sampled vineyards ranged from 37 to 820 km (Supplementary Table S1). Vineyard information and details of QoI applications during the season are listed in Table 1.

**TABLE 1:** Site of collection, host, vineyard age, details of QoI applications in the vineyard, sampling date and number of isolates collected in Brazilian *Plasmopara viticola* populations analyzed in this study.

Population	Location^a^ (municipality, state)	Host (species and cultivar)	Age (years)	QoI applications^b^	Sampling date	No. of isolates
PPv1	Piracicaba, SP	*Vitis labrusca* ‘Niagara Rosada’	11	–	11/22/2018	24
PPv2	São Roque, SP	*V. vinifera* ‘Cabernet Franc’	7	11/20/2018 – fenamidone	01/21/2019	24
				11/26/2018 – fenamidone		
				01/04/2019 – azoxystrobin		
				01/10/2019 – kresoxim-methyl		
				01/14/2019 – kresoxim-methyl		
				01/16/2019 – kresoxim-methyl		
PPv3	Flores da Cunha, RS	*V. vinifera* ‘Pinot Noir’	11	10/28/2018 – pyraclostrobin	02/13/2019	23
				11/15/2018 – fenamidone		
				12/14/2018 – fenamidone		
				01/05/2019 – pyraclostrobin		
				01/17/2019 – pyraclostrobin		
PPv4	Bento Gonçalves, RS	*V. labrusca* ‘Isabel’	40	–	02/14/2019	23

*^*a*^RS, Rio Grande do Sul State; SP, São Paulo State. ^*b*^Details of QoI applications, including date and fungicide active ingredient treatment, during the 2018/19 growing season. All QoI applications were performed in mixture with at least a fungicide belonging to a different mode of action (carboxylic acid amides and dithiocarbamates). Populations PPv1 and PPv4 were collected from vineyards under organic production system without QoI applications.*

In each vineyard, symptomatic leaves were collected randomly at a minimum distance of 10 m from each other and placed in individual plastic bags for transport. Upon arrival at the laboratory, each leaf was placed on wet filter paper in a moist plastic box (11 × 11 × 3.5 cm) and incubated at 22°C with a 16 h photoperiod for 24 h to stimulate downy mildew sporulation. Then, sporangia from a single-lesion were collected using a sterile needle, suspended in 150 μL sterile distilled water at 4°C and inoculated by depositing 15 droplets (10 μL each) onto the abaxial surface of a healthy leaf of *Vitis vinifera* cv. Cabernet Sauvignon grown in a greenhouse. Inoculated leaves were placed in Petri dishes (150 mm) containing 1% water-agar (WA, Difco Laboratories, United States) amended with streptomycin sulfate (0.5 g L^–1^) and incubated in the dark at 22°C for 24 h. Then, Petri dishes were opened in the laminar-flow hood for 2 h to allow any remaining droplets to evaporate from the leaves, and then they were incubated at 22°C with a photoperiod of 16 h. Successive subcultures were performed at 7 to 10-day intervals during the whole study.

### DNA Extraction

For each isolate, three downy mildew lesions excised with a 10-mm cork borer were ground in liquid nitrogen, and DNA was extracted using a modified cetyl trimethyl ammonium bromide (CTAB) method (Lo Piccolo et al., 2012). The genomic DNA was resuspended in 40 μL of Tris-EDTA (TE) buffer (5 mM) and stored at −20°C.

### Sequencing of Cytochrome *b* Gene and Haplotype Relationship Analysis

The partial cytochrome *b* gene of all isolates was amplified using primers CB 279F (Chen et al., 2007) and StrobiR (Burbidge et al., 2002). Amplifications were performed in a total volume of 25 μL containing 2 μL of DNA (30 ng μL^–1^), 8.5 μL of nuclease-free water, 1 μL of each primer (10 μM) and 12.5 μL of GoTaq Colorless Master Mix (Promega, United States) in a Bio-Rad T100 thermal cycler (Bio-Rad, United States). Amplification conditions were 94°C for 1 min; followed by 40 cycles at 94°C for 1 min, 55°C for 1 min and 72°C for 1 min; with a final DNA extension at 72°C for 8 min. PCR products were visualized on 2% agarose gels stained with SYBR Safe (Invitrogen, United States), and purified using the Wizard SV Gel and PCR Clean-Up System kit (Promega), following the manufacturer’s instructions. PCR products were sequenced on an ABI 3130xl Genetic Analyzer (Applied Biosystems, United States) using the PCR primers. Sequences were assembled, aligned and analyzed with Geneious v.10.0.2 (Kearse et al., 2012).

A TCS haplotype network (Clement et al., 2002), based on cytochrome *b* gene sequences, was constructed in PopART v.1.7 (Leigh and Bryant, 2015) in order to visualize genealogical relationships among Brazilian haplotypes found in our study and haplotypes identified in Europe and North America (United States) by Chen et al. (2007). Cytochrome *b* haplotypes present in our dataset were identified using DnaSP v.5.10.1 (Librado and Rozas, 2009).

### Azoxystrobin Sensitivity Assay

A detached leaf assay was used to determine the sensitivity of 14 representative *P. viticola* isolates, selected based on amino acid variation in the cytochrome *b* gene, to the QoI fungicide azoxystrobin. The commercial formulation of azoxystrobin (Amistar WG, 50% active ingredient, Syngenta) was used at the following concentrations: 0, 0.001, 0.01, 0.1, 1, 10, and 100 μg mL^–1^. Leaves from the fourth through the sixth node distal from the shoot tip of potted plants of ‘Cabernet Sauvignon’ grown in a greenhouse were excised, superficially disinfected for 1 min in 0.5% sodium hypochlorite and triple rinsed in sterile distilled water. Subsequently, the leaves were dried at room temperature and then soaked in fungicide solution for 1 min. Leaves were placed with abaxial surface facing upward in 150-mm Petri dishes containing 1% WA with streptomycin sulfate (0.5 g L^–1^). Fresh sporangia were harvested from 7-day-old cultures into sterile distilled water at 4°C, and the suspension was adjusted to 5 × 10^4^ sporangia mL^–1^. Each detached leaf was inoculated by placing 10 droplets (10 μL each), evenly distributed, on the abaxial surface and incubated at 22°C in the dark for 24 h. Thereafter, the Petri dish lids were removed for 2 h to allow the leaf surface to dry, and then they were incubated at the same temperature under a 16 h photoperiod. At 7 days after inoculation, the sporulation in each droplet was visually estimated using the following scale: 0 = no sporulation; 1 = light sporulation; 2 = the sporulation spot was smaller than the inoculum droplet; 3 = the sporulation spot was the same size as the inoculum droplet and; 4 = the sporulation spot was larger than the initial inoculum droplet (Genet et al., 1997). Three replicates for each isolate–fungicide concentration combination were performed, with each replicate consisting of one detached leaf. The average score for each fungicide concentration was converted to a percentage of inhibition by comparison with zero fungicide control. The effective fungicide concentration at which sporulation was inhibited by 50% (EC_50_) was determined by linear regression analysis of the sporulation inhibition percentage versus log_10_-transformed fungicide concentration using Microsoft Excel software. The minimum inhibitory concentration (MIC), the lowest concentration able to completely inhibit *P. viticola* growth, was also determined. The experiment was performed twice and the combined data demonstrated that variances were homogeneous according Levene’s test (*P* > 0.05). Levene’s test was performed using the package *lawstat* v.3.3 (Gastwirth et al., 2019) in R v.3.6.1 (R Core Team, 2019).

### Microsatellite Genotyping

*Plasmopara viticola* isolates were genotyped with 10 microsatellite loci: CES, ISA (Gobbin et al., 2003 modified by Matasci et al., 2010), Pv7, Pv17, Pv31 (Delmotte et al., 2006), Pv61, Pv137, Pv140, Pv144, and Pv147 (Rouxel et al., 2012) (Supplementary Table S2). Forward primers were labeled with one of four fluorescent dyes: 6-FAM, NED, PET or VIC. PCRs were carried out separately for each microsatellite locus in a volume of 12.5 μL containing 2 μL of DNA (30 ng μL^–1^), 3.25 μL of nuclease-free water, 0.5 μL of each primer (10 μM) and 6.25 μL GoTaq Colorless Master Mix (Promega). PCR amplification was performed as follows: 94°C for 4 min followed by 38 cycles of 30 s at 94°C, 30 s at 54°C, and 35 s at 72°C; and a final extension at 72°C for 5 min. After amplification, the PCR products were multiplexed in two sets according to their size and fluorescent dye attached to the primer (Supplementary Table S2). Combined PCR products were separated by capillary electrophoresis on an ABI 3500 Genetic Analyzer (Applied Biosystems) using GeneScan^TM^ 600 LIZ^®^ (Applied Biosystems) as an internal size standard. Allele sizes were scored using GeneMarker v.3.0.1 (Hulce et al., 2011). Statistical binning of the alleles into fragment size classes, according to the repeat units at each locus, was performed using FlexiBin (Amos et al., 2007) (Supplementary Table S3).

### Genetic Diversity and Mode of Reproduction

A genotype accumulation curve was built to assess whether the 10 microsatellite loci were able to capture the genetic diversity in the dataset using the R package *poppr* v.2.8.3 (Kamvar et al., 2014). The genetic diversity of *P. viticola* was assessed at four different levels, grouping isolates according to: (*i*) vineyard population (PPv1-4); (*ii*) production system (conventional and organic); (*iii*) QoI sensitivity (sensitive and resistant, based on the absence/presence of the G143A mutation, respectively) and; (*iv*) state of origin (Rio Grande do Sul and São Paulo). We used GenAlEx v.6.503 software (Peakall and Smouse, 2012) to identify distinct multilocus genotypes (MLGs), where a single difference in an allele size was considered enough to discriminate a unique MLG. Clonal fraction was calculated as 1 – (number of different MLGs/total number of isolates). Number of alleles (*N*_A_), number of private alleles and unbiased Nei’s gene diversity (*H*; Nei, 1978) were also determined using GenAlEx. Allelic richness (*A*_R_) was estimated using the rarefaction method to account for variable population sizes in FSTAT v.2.9.4 (Goudet, 2003) based on 1,000 permutations. Genotypic evenness (*E*_5_) and genotypic diversity including Stoddart and Taylor (*G*) and Simpson (λ) indices were calculated in *poppr*. *G* is a genotypic diversity index based on sample size (Stoddart and Taylor, 1988), while λ is unbiased to sample size (Simpson, 1946).

To investigate the reproduction mode of *P. viticola* at vineyard level, the index of association (*I*_A_) and the standardized index of association (*r*_d_) were calculated using clone-corrected data in *poppr*. The *I*_A_ is a multilocus measure of linkage disequilibrium based on the distribution of the number of different alleles between all pairs of individuals (Milgroom, 2015). As *I*_A_ is dependent on the number of loci assayed, we also calculated the *r*_d_ that removes this dependency (Agapow and Burt, 2001). Departure from the null hypothesis of random association of alleles, consistent with random mating (*I*_A_ and *r*_d_ = 0), was assessed by 999 permutations. Loci were tested to determine whether they were under Hardy–Weinberg equilibrium in the R package *pegas* v.0.11 (Paradis, 2010) and significance was calculated by 999 Monte Carlo permutations.

### Population Structure

The genetic population structure of *P. viticola* was analyzed by discriminant analysis of principal components (DAPC) using the R package *adegenet* v.2.1.1 (Jombart, 2008). DAPC is a multivariate method designed to identify and describe groups/clusters of genetically related individuals based on data transformation using a principal components analysis (PCA) and subsequently clusters are identified using discriminant analysis (DA) (Jombart et al., 2010). At first, we used the ‘find.cluster’ function to determine the optimal number of genetic clusters (*K*) in the dataset using the Bayesian Information Criterion (BIC) without prior information on group membership. The optimal number of principal components retained for the DAPC was determined by cross-validation using the ‘xvalDapc’ function, with a training set consisting of 90% of the data and then used to predict the groups of the 10% of remaining observations (Jombart et al., 2010). After running the DAPC, the probabilities of assignment of each MLG to the different genetic clusters were plotted using the ‘compoplot’ function. To visualize the phylogenetic relationships among MLGs and populations, as well as to identify the prevalence of each MLG across populations, a minimum spanning network (MSN) was performed in *poppr* using Bruvo’s distance (Bruvo et al., 2004).

The pairwise fixation index (*F*_ST_) was used to estimate the genetic differentiation between populations in Arlequin 3.5.2.2 (Excoffier and Lischer, 2010), with significance assessed by 10,000 permutations. *F*_ST_ values <0.05 indicate little genetic differentiation, 0.05–0.15 moderate, 0.15–0.25 great, and >0.25 very great genetic differentiation (Wright, 1978). Analysis of molecular variance (AMOVA) was carried out to determine the distribution of genetic variation between and within populations in *poppr*. AMOVA was performed using a pairwise matrix of Bruvo’s distance calculated with the ‘bruvo.dist’ function, and significance was tested using 999 permutations. Both AMOVA and pairwise *F*_ST_ analyses were conducted using clone-corrected data at different levels.

## Results

### Sequencing of Cytochrome *b* Gene and Haplotype Relationship Analysis

**FIGURE 1:**
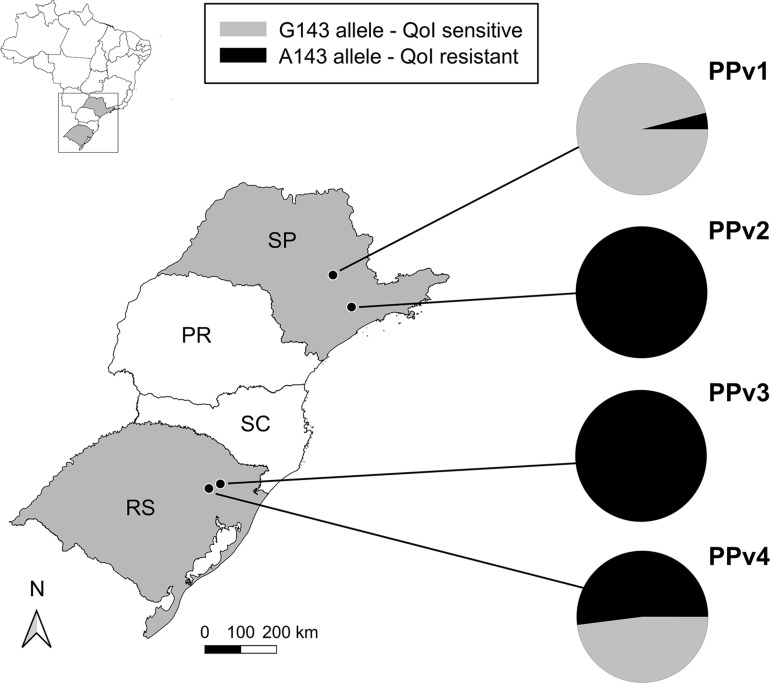
Distribution and QoI sensitivity of *Plasmopara viticola* isolates sampled in vineyards in the 2018/19 growing season in Brazil. The PPv1 and PPv4 vineyard populations were collected from organic vineyards and PPv2 and PPv3 were sampled from conventional vineyards. Pie charts show the relative frequencies of isolates carrying either the G143 (QoI sensitive – gray color) or the A143 (QoI resistant – black color) alleles in the cytochrome *b* gene in each population. PR, Paraná; RS, Rio Grande do Sul; SC, Santa Catarina; and SP, São Paulo States.

A 537 bp partial fragment of the cytochrome *b* gene was amplified and sequenced for all 94 *P. viticola* isolates collected from vineyards under organic (PPv1 and PPv4) and conventional (PPv2 and PPv3) production systems. A total of five polymorphic sites were identified in the dataset. However, only two of them were non-synonymous mutations resulting in the replacement of methionine [M (ATG)] by isoleucine [I (ATA)] at codon 125 (M125I) and glycine [G (GGT)] to alanine [A (GCT)] at codon 143 (G143A). The M125I alteration was found only in isolates from PPv4 (13%). All isolates from PPv2 and PPv3, 4% from PPv1 and 52% from PPv4 carried the G143A mutation (Figure 1).

**FIGURE 2:**
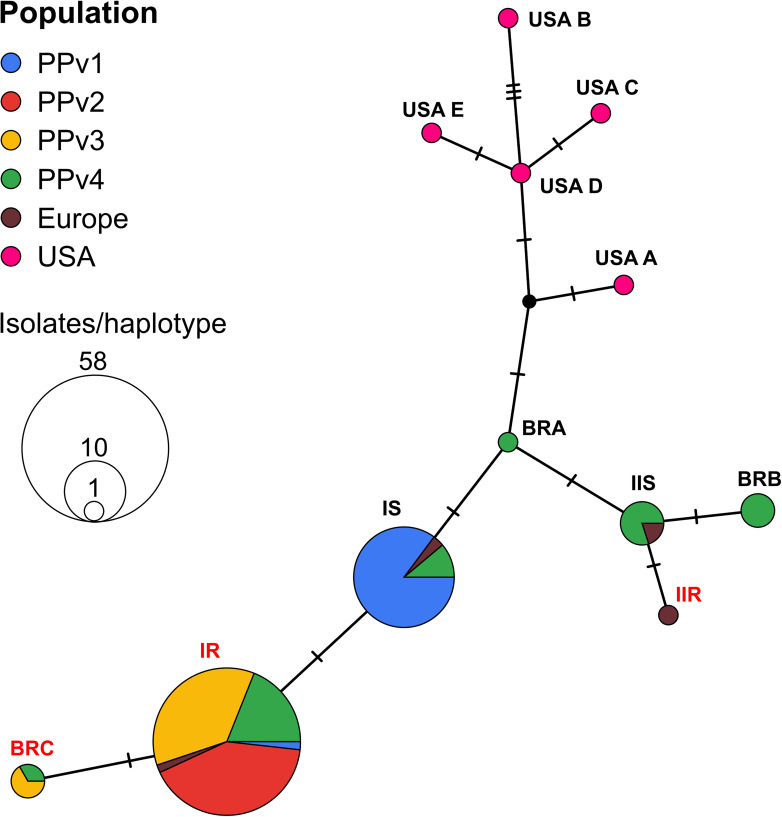
TCS haplotype network of *Plasmopara viticola*, based on partial cytochrome *b* gene sequences, showing the genealogical relationships among the haplotypes. Each circle represents distinct haplotypes, proportional in size to its frequency in the sample. Small black dot indicates hypothetical haplotype not detected in the data set. Each short black hashed line indicates a single nucleotide polymorphism between each haplotype. Geographic origin of isolates from each haplotype is proportionally represented in pie charts by different colors as shown by the key in the upper left corner. Haplotypes are named as proposed by Chen et al. (2007) and new haplotypes detected in this study were named as BRA, BRB and BRC. Isolates from haplotypes IR, IIR, and BRC (highlighted in red) had the G143A mutation. Haplotype BRB carried the M125I alteration.

The haplotype network analysis revealed the existence of six haplotypes in Brazilian populations, three of them previously reported in the European population (IR, IS, and IIS). The remaining three new haplotypes, never reported before, were identified in the population PPv4 (BRA and BRB) and in both PPv3 and PPv4 (BRC). Among all Brazilian sequences analyzed, the frequency of haplotypes IR, IS, IIS, BRB, BRC, and BRA was approximately 61, 28, 4, 3, 3, and 1%, respectively (Figure 2). Haplotypes IR and BRC carried the G143A mutation, while haplotype BRB had the M125I alteration. No North American haplotypes were detected in Brazilian isolates. A representative sequence of each haplotype identified in this study was deposited in GenBank (accession numbers MT329677–MT329682).

### Azoxystrobin Sensitivity Assay

Three azoxystrobin sensitivity levels were identified among the 14 Brazilian isolates tested (Table 2). The first group formed by wild-type isolates had EC_50_ and MIC values of 0.02 and 0.1 μg mL^–1^, respectively. Isolates carrying the M125I alteration had a five-fold increase in EC_50_ compared to wild-type isolates and a MIC of 1 μg mL^–1^. High resistance levels were observed in isolates carrying G143A (EC_50_ and MIC > 100 μg mL^–1^).

**TABLE 2:** Azoxystrobin sensitivity of *Plasmopara viticola* isolates and cytochrome *b* amino acid substitutions.

Isolate^a^	EC_50_^b^ (μg mL^–1^)	MIC^c^ (μg mL^–1^)	Amino acid at codon 125	Amino acid at codon 143
PPv1.2	0.02	0.1	Met	Gly
PPv1.6	0.02	0.1	Met	Gly
PPv1.14	0.02	0.1	Met	Gly
PPv1.19	0.02	0.1	Met	Gly
PPv4.11	0.12	1	Ile	Gly
PPv4.21	0.10	1	Ile	Gly
PPv2.3	>100	>100	Met	Ala
PPv2.4	>100	>100	Met	Ala
PPv2.10	>100	>100	Met	Ala
PPv3.2	>100	>100	Met	Ala
PPv3.15	>100	>100	Met	Ala
PPv3.21	>100	>100	Met	Ala
PPv4.3	>100	>100	Met	Ala
PPv4.19	>100	>100	Met	Ala

*^*a*^Prefix “PPv” followed by the first number (1, 2, 3, or 4) indicates the population where the isolate was collected. PPv1 and PPv4 were sampled in organic vineyards and PPv2 and PPv3 in conventional vineyards. ^*b*^Effective fungicide concentration at which *P. viticola* sporulation was inhibited by 50%. ^*c*^Minimum inhibitory concentration at which no *P. viticola* growth was observed.*

### Genetic Diversity and Mode of Reproduction

The genotype accumulation curve tended to a plateau from the 7th microsatellite, indicating that the 10 microsatellite loci had enough power to discriminate a significant number of MLGs (Supplementary Figure S1). All 10 microsatellite loci were polymorphic and 61 alleles were identified in total (Supplementary Table S4). The number of alleles per locus ranged from three (Pv7 and Pv147) to 14 (CES). Private alleles were present in all populations except PPv2. A total of 53 MLGs were identified within the entire dataset, and only one genotype, MLG7, was shared between the populations PPv1 and PPv2. The number of MLGs per vineyard population ranged from three to 23 and was highly related to the state of origin (Table 3). Nine MLGs were identified in São Paulo and 44 in Rio Grande do Sul, resulting in a clonal fraction of 0.81 and 0.04, respectively. Analyses at the different levels revealed that *H* and *A*_R_ measures varied from 0.35 to 0.60 and 1.78 to 5.82, respectively. Overall, measures of genetic diversity showed small or no differences between populations within the production system and QoI sensitivity levels. When considering the state of origin, the Rio Grande do Sul cluster of populations had higher genotypic diversity (*G* = 40.69; λ = 0.98) than that from São Paulo (*G* = 2.98; λ = 0.66). Genotype abundance was also higher in Rio Grande do Sul (*E*_5_ = 0.95), if compared to São Paulo (*E*_5_ = 0.63), indicating that most genotypes occur at the same frequency.

**TABLE 3:** Measures of genetic diversity in *Plasmopara viticola* populations in Brazil at different levels.

Population level	*N*^a^	MLGs^b^	Clonal fraction^c^	*N*_A_^d^	*A*_R_^e^	*H*^f^	*G*^g^	λ^h^	*E*_5_^i^
**Vineyard**									
PPv1	24	7	0.71	31	3.09	0.41	2.38	0.58	0.52
PPv2	24	3	0.88	18	1.78	0.36	1.40	0.29	0.56
PPv3	23	21	0.09	38	3.73	0.35	18.24	0.95	0.91
PPv4	23	23	0	55	5.26	0.52	23.00	0.96	1
**Production system**									
Conventional	47	24	0.49	39	3.87	0.45	5.03	0.80	0.39
Organic	47	30	0.36	59	5.82	0.60	8.34	0.88	0.43
**QoI sensitivity**									
Resistant	60	36	0.40	51	4.53	0.49	7.32	0.86	0.36
Sensitive	34	18	0.47	51	5.10	0.55	4.66	0.79	0.43
**State of origin**									
Rio Grande do Sul	46	44	0.04	57	5.59	0.45	40.69	0.98	0.95
São Paulo	48	9	0.81	32	3.18	0.59	2.98	0.66	0.63
**Total**	94	53	0.44	61	–	0.58	10.72	0.91	0.39

*^*a*^Number of isolates in the population. ^*b*^Number of distinct multilocus genotypes. ^*c*^Clonal fraction = 1 − (MLGs/*N*). ^*d*^Number of distinct alleles. ^*e*^Allelic richness with rarefaction to: 19, 43, 34, 41, and 89 individuals for vineyard population, production system, QoI sensitivity, state of origin and total levels, respectively. ^*f*^Unbiased Nei’s gene diversity. ^*g*^Stoddart and Taylor’s genotypic diversity index. ^*h*^Simpson’s genotypic diversity index. ^*i*^Genotypic evenness.*

The indices of association (*I*_A_ and *r*_d_) were not statistically different from zero for populations from Rio Grande do Sul (PPv3 and PPv4), which is expected under a scenario of random mating (Table 4). Unlike, the indices provided evidence of linkage disequilibrium in PPv1 (*P* = 0.001), indicating that asexual reproduction is preponderant in this population from São Paulo. The number of loci under Hardy–Weinberg equilibrium was four, eight and nine for PPv1, PPv3, and PPv4, respectively. Those estimates were not calculate for PPv2 due to its small population size after clone correction (only three distinct MLGs), which could result in loss of power to reject the null hypothesis (Milgroom, 2015).

**TABLE 4:** Index of association (*I*_A_), standardized index of association (*r*_d_) and number of loci in Hardy–Weinberg equilibrium (HWE) using clone-corrected data of *Plasmopara viticola* populations from Brazil.

Population	*N*^a^	MLGs^b^	*I*_A_	*P-*value	*r*_d_	*P-*value	Loci under HWE
PPv1	24	7	4.81	0.001	0.62	0.001	4/10
PPv2	24	3	–	–	–	–	–
PPv3	23	21	0.27	0.087	0.04	0.080	8/10
PPv4	23	23	0.27	0.048	0.03	0.046	9/10

*^*a*^Number of isolates in the population. ^*b*^Number of distinct multilocus genotypes. Significance of *I*_*A*_ and *r*_*d*_ (*P* < 0.01; Taylor et al., 2019).*

### Population Structure

**FIGURE 3:**
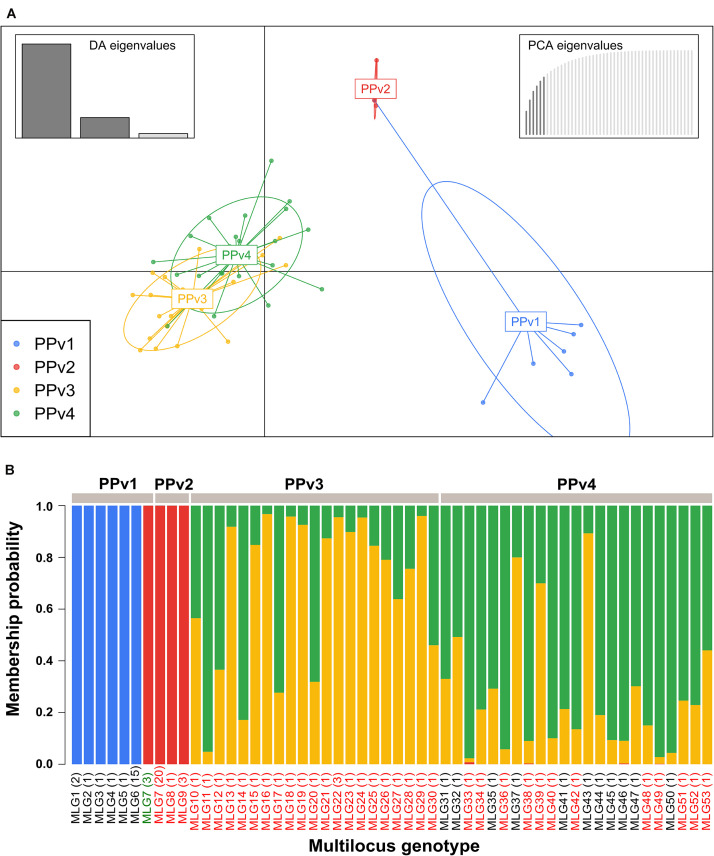
Genetic structure of Brazilian *Plasmopara viticola* populations based on discriminant analysis of principal components (DAPC) using 10 microsatellite loci. **(A)** Scatterplot showing the genetic structure of the four populations displayed by different colors and each dot represents a multilocus genotype (MLG). Inertia ellipse around each population provides graphical summary of a cloud of points. Inset bar plots represent the discriminant analysis (DA) and principal component analysis (PCA) eigenvalues in the upper right and left corners, respectively. **(B)** Membership plot showing the posterior probability assignment of individuals to each genetic cluster (*K* = 4). Colors in each vertical bar represent the probability of a sampled multilocus genotype (MLG) to belong to a genetic cluster. Names of MLGs containing isolates carrying the G143 (QoI sensitive) and A143 (QoI resistant) alleles in the cytochrome *b* gene are shown in black and red, respectively. MLG7 from PPv1 containing two QoI-sensitive isolates and one QoI-resistant isolate is shown in green. The number of isolates of each genotype is indicated in parentheses.

**FIGURE 4:**
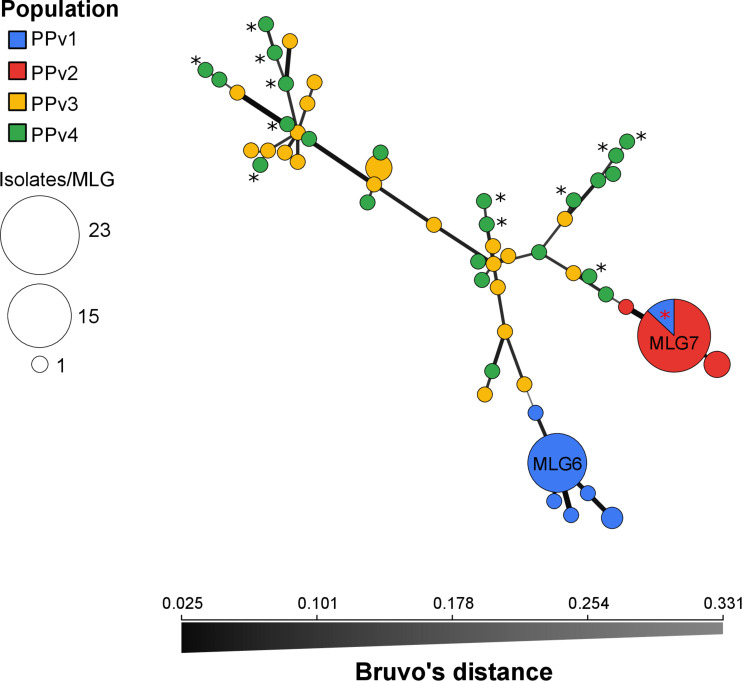
Minimum spanning network of *Plasmopara viticola* populations from Brazil describing the relationship among multilocus genotypes (MLGs). Each MLG is represented by one node sized in proportion to its frequency in the sample and node colors represent population membership. All MLGs from populations PPv2 and PPv3 had the G143A mutation in the cytochrome *b* gene and MLGs from PPv4 carrying the A143 allele are highlighted by black asterisk. MLG7 from PPv1 containing two QoI-sensitive isolates and one QoI-resistant isolate is highlighted by red asterisk. Color intensity and thickness of lines connecting nodes are inversely proportional to Bruvo’s distance.

The BIC revealed that the optimal number of genetic clusters that best described the dataset was *K* = 4 (Supplementary Figure S2). DAPC analysis showed a considerable overlap between the PPv3 and PPv4 populations from Rio Grande do Sul. The first discriminant axis, which explained 79.1% of the variance, separated the populations from Rio Grande do Sul and São Paulo, indicating strong differentiation between states (Figure 3A). The second discriminant axis, representing 17.2% of the discriminatory power, indicated a separation among all but one genotype (MLG7) from PPv1 and PPv2. Posterior assignment probabilities suggested that the presence of MLG7 in PPv1 could be a result of migration from PPv2 (Figure 3B). High levels of genetic admixture were observed in the populations from Rio Grande do Sul, whereas no admixed genotype was identified in the populations from São Paulo. The MSN showed two distant genetic groups present in São Paulo, formed predominantly by the genotypes MLG6 (*n* = 15 isolates from PPv1) and MLG7 (*n* = 3 from PPv1 and *n* = 20 from PPv2) (Figure 4). Meanwhile, MLGs from the Rio Grande do Sul populations were intermixed. No evidence of clustering by QoI sensitivity and production system was found in DAPC and MSN analyses.

Pairwise *F*_ST_ analyses showed significant genetic differentiation between populations at all levels (Supplementary Table S5). At vineyard level, little genetic differentiation was observed between populations PPv3 and PPv4 (*F*_ST_ = 0.04) and great to very great differentiation in other pairwise comparisons (*F*_ST_ = 0.20 to 0.44). Moderate differentiation was detected among isolates grouped by production system (*F*_ST_ = 0.05) and QoI sensitivity (*F*_ST_ = 0.08). Very great genetic difference was observed between the São Paulo and Rio Grande do Sul populations (*F*_ST_ = 0.27). The AMOVA results confirmed these findings and showed that 28.83% of the genetic variation could be explained by differences among populations at vineyard level and 31.82% between populations with respect to the state of origin (Table 5). For production system and QoI sensitivity levels, less than 9% of the variation was found between populations.

**TABLE 5:** Analysis of molecular variance (AMOVA) for clone-corrected *Plasmopara viticola* populations in Brazil at different levels based on Bruvo’s genetic distance.

Population level	Source of variation	Degrees of freedom	Sum of squares	Mean sum of squares	Percentage of variation	*P-*value
Vineyard	Among populations	3	2.26	0.75	28.83	0.001
	Within populations	50	6.59	0.13	71.17	
	Total	53	8.85	0.17	100	
Production system	Between populations	1	0.46	0.47	6.61	0.002
	Within populations	52	8.39	0.16	93.39	
	Total	53	8.85	0.17	100	
QoI sensitivity	Between populations	1	0.54	0.54	8.90	0.001
	Within populations	52	8.31	0.16	91.10	
	Total	53	8.85	0.17	100	
State of origin	Between populations	1	1.17	1.17	31.82	0.001
	Within populations	51	7.49	0.15	68.18	
	Total	52	8.66	0.17	100	

## Discussion

Quinone outside inhibitor resistance in *P. viticola* was reported for the first time in Brazil at a single fungicide trial site in 2000 by a quantitative DNA-based assay detecting cytochrome *b* G143A (Sierotzki et al., 2008). Only many years later, sequencing of partial cytochrome *b* gene of 15 bulk isolates collected from 2014 to 2016 revealed that 11 of them carried the G143A mutation (Moraes, 2016). Although both studies detected the presence of the G143A mutation, no QoI sensitivity data have been published for Brazilian isolates. In this study, pheno- and genotyping assays showed that all azoxystrobin-resistant isolates analyzed (EC_50_ > 100 μg mL^–1^) carried A143 cytochrome *b* alleles, while highly sensitive isolates (EC_50_ = 0.02 μg mL^–1^) carried G143 alleles (Table 2). Surprisingly, a five-fold shift in azoxystrobin sensitivity (mean EC_50_ = 0.11 μg mL^–1^) was observed in a few isolates harboring the M125I alteration, which was never reported before, to the best of our knowledge. M125 is located within the heme *b*_1_ distal lobe of the QoI binding site formed by amino acid residues 120–150 close to the surface region of cytochrome *b* (Fisher et al., 2004). Alterations affecting QoI binding have been described at nearby residues in *Saccharomyces cerevisiae* (A126T) and many plant pathogens, including *P. viticola* (F129L) (Fisher and Meunier, 2008). Interestingly, I125 is naturally present in *S. cerevisiae* and is therefore not likely associated with a fitness cost (Fisher et al., 2004). Thus, further investigations are needed to evaluate the effect of the M125I alteration on sensitivity to other QoI active ingredients. Similarly, the new described V204I and V325I alteration in the cytochrome *b* gene of *Pythium paroecandrum* were associated with a 30-fold reduction (EC_50_ > 14.6 μg mL^–1^) in azoxystrobin sensitivity compared to wild-type isolates (Matic et al., 2019). However, the slight loss of sensitivity apparently associated with the M125I alteration should not affect field performance of azoxystrobin against *P. viticola*. In most pathosystems, practical control failures have been associated with the G143A mutation, while the F129L and G137R mutations confer partial resistance that is usually overcome by the recommended field doses (Fernández-Ortuño et al., 2008).

Cytochrome *b* sequencing revealed that all isolates analyzed from vineyards under conventional management had the G143A mutation (Figure 1), indicating that appropriate resistance management should be undertaken to avoid disease control failure. Further investigations are needed to quantify the extent of resistance in the vineyards to suggest the appropriate resistance management to the grape-growers. Monitoring data suggest that a few QoI applications can result in a strong selection pressure in *P. viticola*, changing drastically the G143A frequency of less than 1 to 100% within a growing season (Sierotzki et al., 2008). However, QoI-resistant populations gradually reverted to full sensitivity after stopping QoI treatments, possibly due to the reduced fitness of QoI-resistant isolates (Sierotzki et al., 2008). According to the Fungicide Resistance Action Committee (FRAC^1^), a maximum of three QoI applications preventively in mixture with effective partners from different cross-resistance groups should be used per vine cycle to avoid resistance development. However, the fungicide partner and its dose in the mixture significantly affect the success of QoI resistance management strategies. For instance, applications of the phthalimide fungicide folpet in mixture with QoIs delayed selection pressure in *P*. *viticola* compared to solo QoI applications, while the mixture with the dithiocarbamate fungicide mancozeb had low efficiency (Genet et al., 2006). All QoI applications in the vineyards sampled in our study were in mixture with dithiocarbamates and/or CAAs, and the low efficiency of dithiocarbamates to avoid QoI resistance development associated with CAA control failures reported by the grape-growers may explain the high frequency of QoI-resistant isolates. In contrast to QoI-treated populations, populations sampled in organic vineyards had a lower number of isolates carrying the G143A mutation (PPv1 = 4% and PPv4 = 52%). The higher frequency detected in PPv4 may be attributed to migrations of QoI-resistant spores from adjacent areas because the vineyard from which this population was sampled is located in a traditional grape-growing area near many QoI-treated vineyards. For instance, 32 alleles were shared among QoI-resistant isolates from PPv3 and PPv4, indicating gene flow between them. In Italy, bulk samples from untreated vineyards showed cytochrome *b* G143A frequencies up to 26%, suggesting that QoI-resistant spores of *P. viticola* are able to disperse among neighboring areas (Toffolatti et al., 2007, 2011).

The haplotype network based on cytochrome *b* gene sequences detected six distinct haplotypes in Brazil, three of which were also found in Europe (Figure 2). The topology was similar to those previously published using North American and European isolates (Chen et al., 2007; Delmas et al., 2017); however, we identified three new haplotypes (BRA, BRB, and BRC) at low frequencies in Rio Grande do Sul. Interestingly, none of the haplotypes previously detected in the North America were found in our studies. Although *P. viticola* was firstly introduced in Brazil in the end of the 19th century by infected propagation material from North America (Sousa, 1996), multiple introductions probably occurred during the 20th century via *V. vinifera* grapevines commonly imported from Europe. Analysis of the global genetic structure of *P. viticola* revealed that the South American population, composed by a few isolates from South-eastern Brazil and Uruguay, was more closely related to the French population than the North American population (Taylor et al., 2019). Similarly, our findings indicate that *P. viticola* from Brazil and Europe are genetically similar and the new haplotypes identified are the result of mutations in the cytochrome *b* gene, since the mitochondrial genome does not recombine. The two major haplotypes IS and IR contained 89% of the isolates tested, and similar results were also reported in European populations (Corio-Costet et al., 2011). Our analysis supports that the G143A mutation has a single origin in the populations tested, represented by the haplotype IR that later originated the haplotype BRC by a single additional mutation. On the other hand, QoI resistance has two independent origins in Europe (Chen et al., 2007; Delmas et al., 2017).

Microsatellite analysis revealed high genetic variability among the four vineyard populations studied; however, small differences in allelic and genotypic diversity were observed between isolates grouped by production system and QoI sensitivity (Table 3). In contrast, we found greater differences when comparing isolates based on their state of origin. For instance, lower clonal fraction (0.04) and higher genotypic diversity (*G* = 40.69) were observed in Rio Grande do Sul compared to São Paulo (clonal fraction = 0.81; *G* = 2.98). A recent study also revealed that *P. viticola* populations from São Paulo had low genetic diversity, where 19 of the 55 MLGs were clonal and represented 93% of the isolates sampled (Camargo et al., 2019). Meanwhile, the high diversity observed in Rio Grande do Sul is similar to that in Europe, where sexual reproduction appears to play a major role in downy mildew epidemics (Gobbin et al., 2006; Delmas et al., 2017). In fact, the indices of association showed that *P. viticola* populations in Rio Grande do Sul are predominantly panmictic, whereas São Paulo populations are largely clonal (Table 4).

The pairwise *F*_ST_ and AMOVA analyses also supported that state of origin influences the population biology of *P. viticola*, while production system and QoI sensitivity have little effect (Table 5 and Supplementary Table S5). Likewise, a little genetic differentiation was found between South African *P. viticola* populations from conventional and organic vineyards assessed in two consecutive growing seasons (Koopman et al., 2007). Differences in downy mildew genetic diversity, population structure and mode of reproduction between isolates from Rio Grande do Sul and São Paulo may be due to climatic conditions that have direct influence on the grapevine phenology and pruning management, as well as pathogen reproduction and survival. The probability of clone survival between seasons in Rio Grande do Sul is very low because cold temperatures during the autumn and winter induce grapevine dormancy and *P. viticola* overwinters as oospores formed within infected leaves on the soil surface, as reported in a preliminary study (Cirne et al., 1988). Conversely, the climate in São Paulo is favorable all year-round for grapevine growth and green tissues are present during almost the entire year due to the double pruning system commonly used in the state. Thus, *P. viticola* reproduces and survives predominantly asexually on green tissues (Camargo et al., 2019). Due to the mild winter in South Africa, *P. viticola* populations often experience bottlenecks at the end of the growing season and a few highly adapted genotypes can survive between seasons as asexual spores or as vegetative mycelium (Koopman et al., 2007). In this scenario, epidemics are driven by clonal infections of these adapted asexual clonal lineages although oosporic infections throughout the season can occur (Koopman et al., 2007). Similarly, the populations from São Paulo were dominated by the clonal genotypes MLG6 and MLG7 that represented 79% of the isolates, while the occurrence of the five single genotypes may be the result of oosporic infections (Figure 4). However, further studies are needed to determine whether oospores are formed and to determine the relative contribution of asexual and sexual inoculum throughout growing seasons in both states.

The very great differentiation between states was confirmed in the DAPC and MSN analyses (Figures 3, 4). Our results indicated that populations from Rio Grande do Sul are admixed between two genetically distinct pools and have a weak, but significant, genetic differentiation, suggesting considerable genetic recombination and gene flow. We believe that these two genetic pools are widely spread over the region sampled because viticulture has been intensively practiced for more than 100 years with frequent exchange of propagation material among grape growers in the past. The recombination through sexual reproduction, as suggested by our results, may allow *P. viticola* to adapt quickly to environmental changes and management practices including fungicides and resistant cultivars (McDonald and Linde, 2002; Milgroom, 2015). In Europe, *P. viticola* populations are also panmictic and admixed from two genetically distinct clusters (Fontaine et al., 2013; Maddalena et al., 2020) and a rapid adaptation to different fungicide groups (Blum et al., 2010; Delmas et al., 2017; Fontaine et al., 2019) and to resistant cultivars (Peressotti et al., 2010) has been reported. Although other two different genetic pools were observed in the São Paulo populations, no admixed genotype was detected, which is consistent with lack of recombination inferred from the reproductive mode analysis. The presence of an identical genotype (MLG7) shared between populations from São Paulo provides evidence for long-distance dispersal, since there was no exchange of propagation material and equipment between the grape-growers, as also reported by Camargo et al. (2019). Among the three isolates belonging to genotype MLG7 in population PPv1, only one carried the G143A mutation. Probably the genotype MLG7 must have been at a very high frequency in the past and G143A did evolve in some clonal isolates of this genetic background. Another hypothesis is that MLG7 may have lost the G143A mutation without selection pressure of QoI in this organic vineyard (PPv1) as there are many G143A isolates of MLG7 in PPv2, where QoIs are often applied.

## Conclusion

The results of the present study showed that the population genetics of *P. viticola* is climate-driven, while production system (conventional or organic) and QoI sensitivity have little or no effect. It seems that the population genetic differences between Rio Grande do Sul and São Paulo are related to the distinct winter weather conditions, which have a profound influence on the reproductive mode and, consequently, in the allelic and genotypic diversity of *P. viticola* populations. As expected, populations from conventional vineyards had higher frequencies of G143A QoI-resistant isolates than those from organic vineyards. A new non-synonymous amino acid alteration at codon 125 in the cytochrome *b*, associated with a slight reduction in azoxystrobin sensitivity, was reported for the first time in *P. viticola*. Moreover, Brazilian *P. viticola* populations are more closely related to the European than the North American population based on cytochrome *b* analysis. Therefore, this study provides a solid support for further epidemiological studies and fungicide resistance management for downy mildew in subtropical regions.

## Data Availability Statement

The datasets presented in this study can be found in online repositories. The names of the repository/repositories and accession number(s) can be found below: https://www.ncbi.nlm.nih.gov/genbank/, MT329677–MT329682.

## Author Contributions

RS, MC-G, LA, and BF designed the experiments. RS performed the experiments. RS and AO analyzed the data. All authors participated in writing and editing the manuscript.

## Conflict of Interest

The authors declare that the research was conducted in the absence of any commercial or financial relationships that could be construed as a potential conflict of interest. The handling Editor declared a shared affiliation, though no other collaboration with several of the authors [RS, MC-G, AO, and LA] at time of review.

## Footnotes

1 www.frac.info
