# Supplementary material for: The Climate-Driven Genetic Diversity Has a Higher Impact on the Population Structure of Plasmopara viticola Than the Production System or QoI Fungicide Sensitivity in Subtropical Brazil
Source: Front Microbiol. 2020 Sep 17;11:575045. doi: 10.3389/fmicb.2020.575045 (PMC7528563; doi:10.3389/fmicb.2020.575045)
Supplement: Supplementary file 1 [file Image_1.pdf]

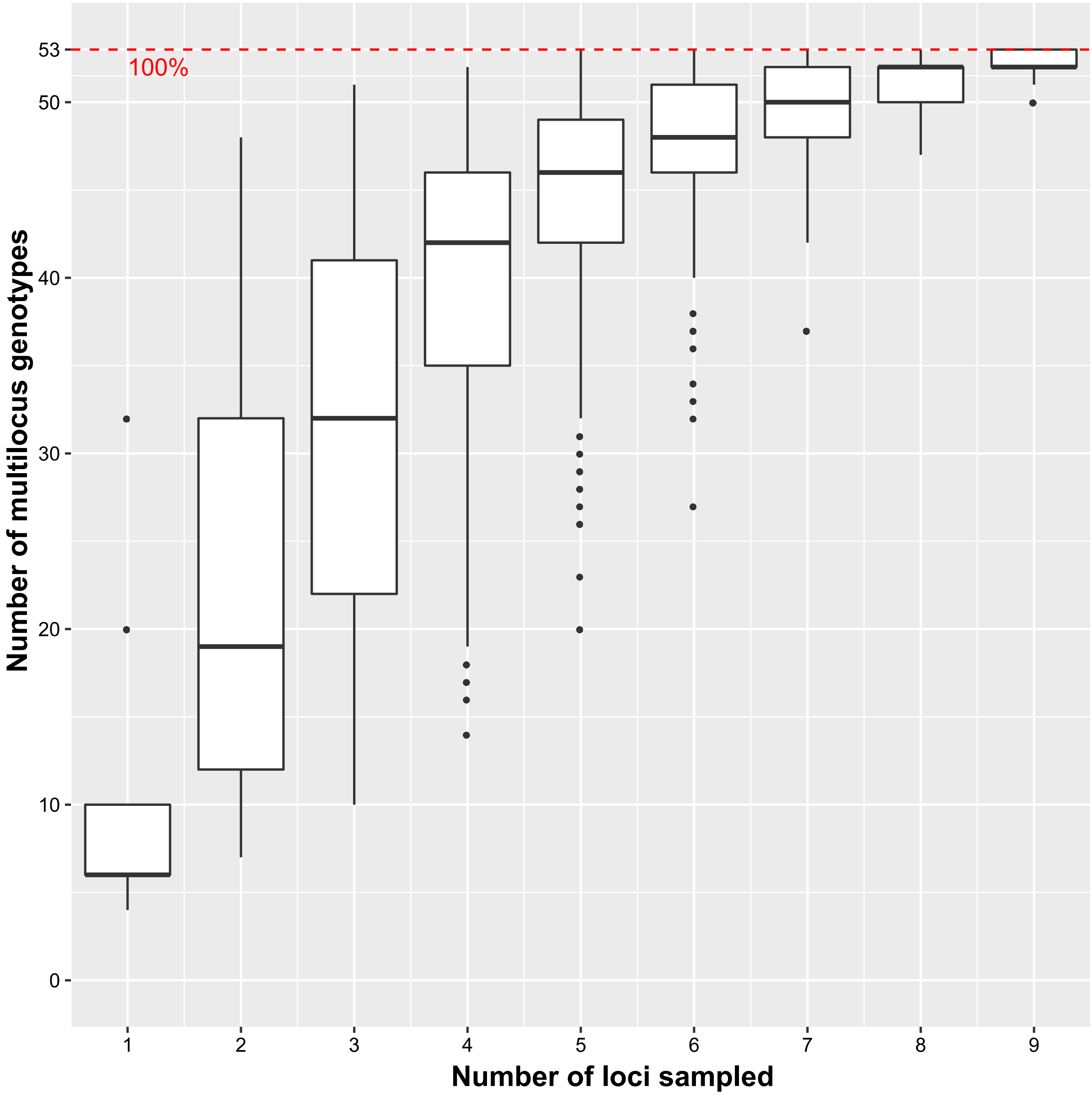

Supplementary Figure S1. Genotype accumulation curve across 10 microsatellite loci for four *Plasmopara viticola* populations from Brazil.
