# Supplementary material for: The Climate-Driven Genetic Diversity Has a Higher Impact on the Population Structure of Plasmopara viticola Than the Production System or QoI Fungicide Sensitivity in Subtropical Brazil
Source: Front Microbiol. 2020 Sep 17;11:575045. doi: 10.3389/fmicb.2020.575045 (PMC7528563; doi:10.3389/fmicb.2020.575045)
Supplement: Supplementary file 2 [file Image_2.pdf]

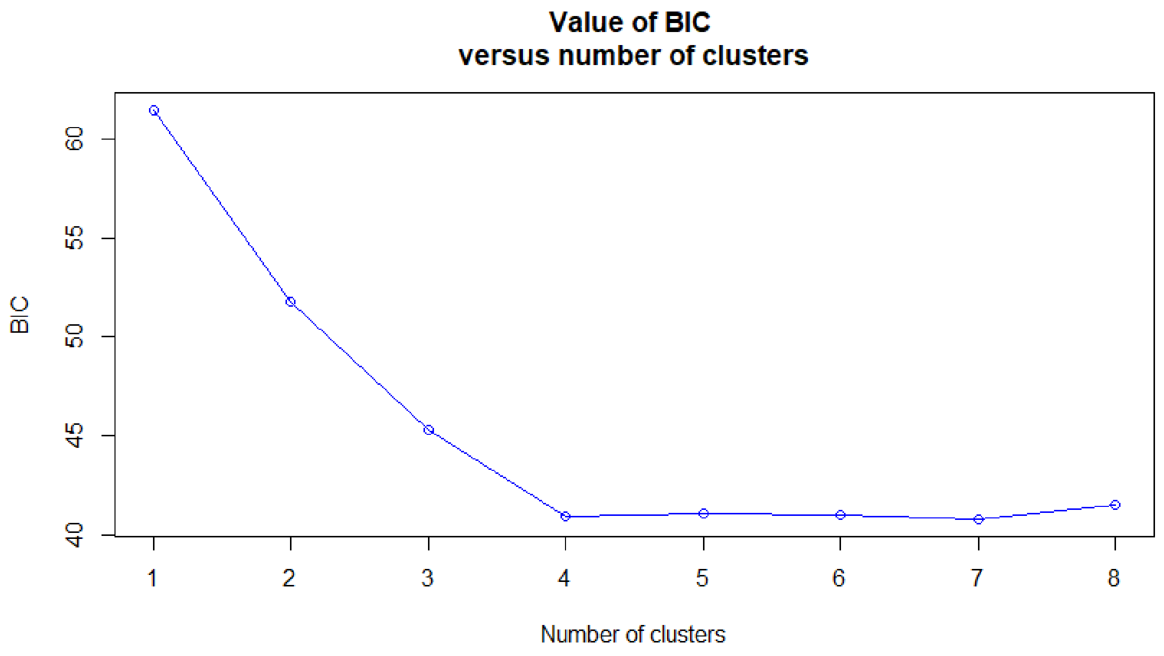

Supplementary Figure S2. Genetic structure of *Plasmopara viticola* populations inferred by discriminant analysis of principal components (DAPC). The lowest Bayesian information criterion (BIC) value was considered as the optimal number of genetic groups/clusters (K) in the dataset ($K = 4$).
