# Supplementary material for: The Climate-Driven Genetic Diversity Has a Higher Impact on the Population Structure of Plasmopara viticola Than the Production System or QoI Fungicide Sensitivity in Subtropical Brazil
Source: Front Microbiol. 2020 Sep 17;11:575045. doi: 10.3389/fmicb.2020.575045 (PMC7528563; doi:10.3389/fmicb.2020.575045)
Supplement: Supplementary file 3 [file Table_1.docx]

**Supplementary Table S1.** Geographic distance (km) among *Plasmopara viticola* populations collected in Brazil.

| **Population** | PPv1 | PPv2 | PPv3 |
| --- | --- | --- | --- |
| PPv2 | 123 |  |  |
| PPv3 | 791 | 724 |  |
| PPv4 | 820 | 756 | 37 |
