# Supplementary material for: The Climate-Driven Genetic Diversity Has a Higher Impact on the Population Structure of Plasmopara viticola Than the Production System or QoI Fungicide Sensitivity in Subtropical Brazil
Source: Front Microbiol. 2020 Sep 17;11:575045. doi: 10.3389/fmicb.2020.575045 (PMC7528563; doi:10.3389/fmicb.2020.575045)
Supplement: Supplementary file 4 [file Table_2.docx]

**Supplementary Table S2.** Characteristics of the microsatellite loci used to estimate genetic diversity in *Plasmopara viticola* populations from organic and conventional vineyards in Brazil.

| **Multiplex**^a^ | **Microsatellite** | **Fluorescent dye** | **Repeat motif** | **Size range (bp)** | **Source** |
| --- | --- | --- | --- | --- | --- |
| 1 | Pv17 | PET | (TC)_12_ | 160–172 | Delmotte et al. (2006) |
|  | Pv137 | 6-FAM | (AT)_9_ | 243–256 | Rouxel et al. (2012) |
|  | Pv144 | VIC | (AT)_12_ | 161–192 | Rouxel et al. (2012) |
|  | Pv147 | NED | (TCGACT)_8_ | 189–219 | Rouxel et al. (2012) |
|  |  |  |  |  |  |
| 2 | CES | VIC | (TC)_n_(AC)_n_ | 143–186 | Matasci et al. (2010) |
|  | ISA | 6-FAM | (TC)_n_ | 118–144 | Matasci et al. (2010) |
|  | Pv7 | PET | (TG)_7_ | 289–297 | Delmotte et al. (2006) |
|  | Pv31 | 6-FAM | (CA)_9_ | 241–247 | Delmotte et al. (2006) |
|  | Pv61 | PET | (CA)_9_ | 181–187 | Rouxel et al. (2012) |
|  | Pv140 | NED | (TA)_9_ | 172–201 | Rouxel et al. (2012) |

^a^ Microsatellites were individually amplified and then PCR products were combined into two multiplex sets for genotyping.
