# Supplementary material for: The Climate-Driven Genetic Diversity Has a Higher Impact on the Population Structure of Plasmopara viticola Than the Production System or QoI Fungicide Sensitivity in Subtropical Brazil
Source: Front Microbiol. 2020 Sep 17;11:575045. doi: 10.3389/fmicb.2020.575045 (PMC7528563; doi:10.3389/fmicb.2020.575045)
Supplement: Supplementary file 6 [file Table_4.docx]

**Supplementary Table S4.** Number of distinct alleles at each microsatellite locus and total number of private alleles in *Plasmopara viticola* collected in Brazil at different levels.

|  |  | **Number of alleles** | | | | | | | | | | **Number of private alleles** |
| --- | --- | --- | --- | --- | --- | --- | --- | --- | --- | --- | --- | --- |
| **Population level** | ***N***^a^ | **Pv7** | **Pv17** | **Pv31** | **Pv61** | **Pv137** | **Pv140** | **Pv144** | **Pv147** | **CES** | **ISA** |  |
| **Vineyard** |  |  |  |  |  |  |  |  |  |  |  |  |
| PPv1 | 24 | 3 | 3 | 4 | 4 | 2 | 4 | 4 | 1 | 3 | 3 | 4 |
| PPv2 | 24 | 1 | 2 | 2 | 2 | 1 | 2 | 3 | 1 | 2 | 2 | 0 |
| PPv3 | 23 | 2 | 3 | 3 | 3 | 4 | 3 | 10 | 1 | 7 | 2 | 2 |
| PPv4 | 23 | 2 | 4 | 4 | 4 | 5 | 7 | 12 | 3 | 11 | 3 | 15 |
|  |  |  |  |  |  |  |  |  |  |  |  |  |
| **Production system** |  |  |  |  |  |  |  |  |  |  |  |  |
| Conventional | 47 | 2 | 3 | 3 | 3 | 4 | 4 | 10 | 1 | 7 | 2 | 2 |
| Organic | 47 | 3 | 4 | 4 | 4 | 5 | 7 | 13 | 3 | 12 | 4 | 22 |
|  |  |  |  |  |  |  |  |  |  |  |  |  |
| **QoI sensitivity** |  |  |  |  |  |  |  |  |  |  |  |  |
| Resistant | 60 | 2 | 3 | 3 | 3 | 5 | 6 | 12 | 3 | 12 | 2 | 10 |
| Sensitive | 34 | 3 | 4 | 4 | 4 | 4 | 7 | 12 | 1 | 8 | 4 | 10 |
|  |  |  |  |  |  |  |  |  |  |  |  |  |
| **State of origin** |  |  |  |  |  |  |  |  |  |  |  |  |
| Rio Grande do Sul | 46 | 2 | 4 | 4 | 4 | 5 | 7 | 12 | 3 | 13 | 3 | 29 |
| São Paulo | 48 | 3 | 3 | 4 | 4 | 2 | 4 | 5 | 1 | 3 | 3 | 4 |
|  |  |  |  |  |  |  |  |  |  |  |  |  |
| **Total** | 94 | 3 | 4 | 4 | 4 | 5 | 7 | 13 | 3 | 14 | 4 | - |

^a^ Number of isolates.
