# Supplementary material for: The Climate-Driven Genetic Diversity Has a Higher Impact on the Population Structure of Plasmopara viticola Than the Production System or QoI Fungicide Sensitivity in Subtropical Brazil
Source: Front Microbiol. 2020 Sep 17;11:575045. doi: 10.3389/fmicb.2020.575045 (PMC7528563; doi:10.3389/fmicb.2020.575045)
Supplement: Supplementary file 7 [file Table_5.docx]

**Supplementary Table S5.** Pairwise *F*_ST_ values for clone corrected *Plasmopara viticola* populations in Brazil at different levels.

| **Population level** |  |  |  |  |
| --- | --- | --- | --- | --- |
| **Vineyard** | PPv1 | PPv2 | PPv3 | PPv4 |
| PPv1 | - |  |  |  |
| PPv2 | 0.41 | - |  |  |
| PPv3 | 0.44 | 0.37 | - |  |
| PPv4 | 0.31 | 0.20 | 0.04 | - |
| **Production system** | Conventional | Organic |  |  |
| Conventional | - |  |  |  |
| Organic | 0.05 | - |  |  |
| **QoI sensitivity** | Resistant | Sensitive |  |  |
| Resistant | - |  |  |  |
| Sensitive | 0.08 | - |  |  |
| **State of origin** | Rio Grande do Sul | São Paulo |  |  |
| Rio Grande do Sul | - |  |  |  |
| São Paulo | 0.27 | - |  |  |

All pairwise *F*_ST_ values were significant (*P* < 0.05).
